# Essential oil composition of *Callistemon citrinus *(Curtis) and its protective efficacy against *Tribolium castaneum *(Herbst) (Coleoptera: Tenebrionidae)

**DOI:** 10.1371/journal.pone.0270084

**Published:** 2022-08-19

**Authors:** Maduraiveeran Ramachandran, Kathirvelu Baskar, Manickkam Jayakumar

**Affiliations:** 1 Department of Zoology, Unit of Applied Entomology, University of Madras, Chennai, Tamil Nadu, India; 2 Department of Ecotoxicology, Ross Lifescience Ltd., Bhosari, Pune, Maharastra, India; Tocklai Tea Research Institute, INDIA

## Abstract

*Tribolium castaneum* is one of the major pests of stored grains which causes extensive damages. To control this insect pest many synthetic chemical pesticides are used. However, continuous usage of synthetic fumigants causes pest resurgence, toxic residues, genetic resistance in pests, environmental contamination and health hazards etc., To avert these problems, essential oils are used as bio-fumigants to control the stored pests. They could act as best alternatives to synthetic fumigant in closed environment. Hence, the present study aimed to evaluate the pesticidal activity of *Callistemon citrinus* oil against *Tribolium castaneum*. GC-MS analysis of *C*. *citrinus* essential oil (EO) showed 10 compounds; among them, the major constituent was eucalyptol (1, 8-cineole) at 40.44%. The lethal concentration (LC_50_) values were 37.05 μL/L (adults) and 144.31 μL/L (larvae) at 24 and 48 hrs respectively. Exposure to *C*. *citrinus* EO significantly reduced the beetle fecundity, ovicidal activity, egg hatchability, larvae survival and emergence of adult. The effect of EO on enzymatic activity of *T*. *castaneum* adults was examined *using* Acetylcholinesterase, α-Carboxylesterase, β-Carboxylesterase, Glutathione-S-Transferase, Acid and Alkaline phosphatase assays. The results indicated that the activity of detoxification enzymes drastically varied when compared with control. This EO had toxicant effects on all stages of the life of *T*. *castaneum*.

## Introduction

Insect infestation on stored grains, pulses, and their processed products is a major problem that results in significant economic losses and reduces the quality as well as the quantity of stored products. Stored grains can be infested by several insect pests that cause severe damage. Storage pests alone damaged 14–17 million tonnes of food grains and nearly 15 insect species were listed as major stored grain pests in India [1]. Among them, *Tribolium castaneum* Herbst 1797 (Coleoptera: **Tenebrionidae**) *was* listed in the major pest category of stored grains, it is predominantly found in tropical countries. Both larvae and adults of *T*. *castaneum* feed on grains, seeds and flour.

Fumigation is an effective method of pest management in stored grains. This method is used to control all stages of insects in stored grains and is cost effective, rapidly kills insects and leaves no residue [2]. The commonly used synthetic fumigants in stored grains are ethylene dichloride, carbon bisulphide, methyl bromide and phosphine etc. However, Methyl bromides has ozone depleting properties [3–5]; insect resistance to phosphine has been documented [6, 7]. Application of fumigants leads to increasing pest resurgence, deleterious effects on beneficial organisms, as well as raising the levels of toxicity [8]. To address these problems, biodegradable plant products have been evaluated for insecticidal properties.

Interestingly, plant leaves and oils have been used as a stored grain protectants. More recently, essential oils (EOs) derived from plants have been receiving more attention. Several studies have shown that plant essential oils are having great potential to control the pests of stored grains [9–12]. EOs have been extracted majorly from the families Myrtaceae, Lauraceae, Umbelliferae, Lamiaceae, Asteraceae and conifers [13, 14].

EO can exhibit toxic repellent, pupicidal, ovicidal, and oviposition deterrent activities against insect pests of stored grains [15–20]. *Callistemon citrinus* (Curtis) belongs to the family Myrtaceae, commonly known as bottle brush. The *C*. *citrinus* essential oil is highly aromatic in nature. Literature survey showed that the essential oil of *C*. *citrinus* possesses insecticidal and repellent activity against *Callosobruchus maculatus*, adulticidal activity against *Rhyzopertha dominica* and also the plant was used as antimicrobial, deodorants, perfumes and antifungal agent [21–24]. *T*. *castaneum* (Red flour beetle) is the major pest of stored products like dry fruit, broken cereals, pulses and flour. Both adult and larvae cause severe damages to the stored products which could reduce the seed viability, loss of nutrition, contamination of grains with toxic microbes and pathogens. Hence the present investigation was aimed to evaluate the toxic effect of *C*. *citrinus* EO against *T*. *castaneum*.

## Materials and methods

### Culture of insect

*T*. *castaneum*, was maintained in Insectarium, Department of Zoology, University of Madras The culture was maintained in wheat flour in a plastic container (35 x 20 cm) at room temperature 28±2˚ C and R.H. of 65–70%. For experiments, the flour was sieved by siever gently; adult and larvae were separated by fine brush without damaging.

### Chemicals

α and β naphthol, α and β naphthyl acetate, 1-chloro-2,4-dinitrobenzene (CDNB), Glutathione Reduced (GSH), 5,5-Dithiobis(2-Nitro Benzoic Acid) extrapure (DTNB), Acetylthiocholine iodide, *p*-nitrophenyl phosphate, *p*-Nitrophenol were used for the present study and were of analytical grade. They were purchased from Sigma-Aldrich, Himedia and Sisco Research Laboratories Pvt. Ltd. (India).

### Essential oil extraction

*C*. *citrinus* (Bottle brush) leaves were collected from the campus of University of Madras (13.01140° N, 80.24006° E). The plant was identified by Muniappan Ayyanar, plant taxonomist, A.V.V.M. Sri Pushpam College (Autonomous), Poondi, Thanjavur (Tamilnadu, India). The oil was extracted from the freshly-collected leaves by hydro-distillation using Clevenger Apparatus with 500 g of leaves and 5000 ml of distilled water.

### GC-MS analysis

Gas chromatography–mass spectrometry (GC-MS) -2010 Plus, GCMS-TQ8040 was used to identify the composition of oil. The parameter used were: Column temperature (60°C) with 1 min holding time, injection (240°C) with 10 minutes holding time, injection mode Split, [GCMS-TQ8040] Ion Source Temp: 230.00°C; Interface Temp.: 280.00°C; Solvent Cut Time: 2.50 min; Detector Gain Mode: Relative to the Tuning Result Detector Gain: 0.94 kV +0.20 kV;[MS Table], Start Time: 2.50min;End Time: 29.00min; Acq. Mode: Q3 Scan; Event Time: 0.300sec; Scan Speed: 1666 Start m/z: 50.00; End m/z: 500.00; sample preparation for injection: 10ppm in ethyl acetate; sample injected volume: 2μL; compound identification method: GCMS Tripple quadrapole (Acq. Mode: Q3 Scan).

### Fumigation toxicity

Fumigation toxicity of *C*. *citrinus* EO was tested in the laboratory by filter paper method at 28 ± 2°C and 60–70% RH [25]. Different ranges of concentrations such as 40, 80, 120, 160, 200, 240, 280, and 320 μl/L were evaluated for fumigation toxicity on adults and larvae, respectively. 2–3 days old adults (10/replicate) and 3^rd^ instar larvae (10/replicate) were released in a glass bottle (7 X 3.5 cm) along with 2 g of wheat flour as feed. The EO was poured on Whatman No. 1 filter paper (2 cm dia) and it was adhered inside the screw cap of 100 mL glass container and then closed. Separate control without EO was maintained. Five replications were made for all treatments and controls. Mortality of adults and larvae was recorded after 3, 6, 9, 12, 24, 36, and 48 hrs of the commencements of treatment. Dead insects were counted; if there was no antennal or leg movement, it was considered dead.

### Repellency test

The larval repellency of EO was measured using the diet impregnation method as described by Stefanazzi et al. [26]. Five grams of feed medium were mixed with different concentrations such as of 5, 10, 15, and 20 μL of EO individually in each Petri plate (10 cm X 1.5 cm). Twenty-five 3^rd^ instar larvae were introduced in each treatment and replicated five times. After 2, 4, 6, 12 and 24 hrs of treatment the number of larvae present in the treated and control diets were counted.

The adult repellency of *C*. *citrinus* was evaluated with the help of glass Y tube olfactometer (Stem and both arms measured about 21 cm and 2.5 cm diameter). Two grams of feed medium were mixed with different concentrations such as 5, 10, 15, and 20 μL of EO individually in vial (25 mL) and attached into one arm of the olfactometer. Medium without EO was used as control and was attached into the other arm of Y-tube. Then fifty freshly-emerged adults were released into the stem of the olfactometer. The number of beetles found in each arm was recorded after 24 hrs [27]. The percentage of repellent activity was calculated using the formula of Karemu et al. [28].


%repellency=(Control–Treated/Control+Treated)x100

### Fecundity and knock-down effect of EO on adult

Ten 4 to 6 days old unsexed adults were released into glass vial (100 mL) with 2 g of wheat flour. Filter paper (Whatman No. 1) discs measuring about 2 cm dia, were impregnated with different concentrations (5, 10, 20 and 30 μL/L) of *C*. *citrinus* EO. After 24 hrs, knocked down adults were counted and the live adults were transferred to new Petri plate with feed. The Petri plate was carefully examined and the number of eggs laid in control and treatments for a period of 2 days was recorded by using compound microscope. Five replications were used for each treatment and the control group.

### Growth inhibition effects

Fifty unsexed adult beetles (4–6 days old) were released in a Petri plate containing 5 g of wheat flour. After 48 hrs, the adult beetles were removed and number of eggs laid in each Petri plate was counted. Subsequently, the filter paper treated with different concentrations (5, 10, 20, 30 μL) of EO was placed on the lid of the Petri plate. Filter paper disc devoid of any volatiles was used as control. The experiments were replicated five times for both treatments and control. The eggs hatched in each Petri plate was recorded daily; the hatched larvae were maintained continuously on wheat flour. The larval survivability and per cent adult emergence (F_1_) were recorded.

### Sample preparation for biochemical studies

Adult insects (2–3 days old) were treated with sub-lethal concentrations (5, 10 and 20 μL/L) of EO as mentioned in fumigation toxicity. The live insects were used in the biochemical analysis which consisted of three replicates. Treated adults (10 individuals for each concentration) were transferred separately and homogenized with 500 μL of ice-cold phosphate buffer (20 mM, pH 7.0) using a Teflon, hand homogenizer to estimate the total protein, esterase, phosphatase, and Glutathione-S-Transferase activities. The homogenates were centrifuged at 15,000 rpm at 4°C for 20 min. and the clear supernatants were stored at -20°C until used. The supernatants were used for both qualitative and quantitative analyses of biochemical studies. The concentration of total protein content was estimated by the method of Bradford [29]. Three replicates were maintained for all the enzymes studies.

### Acetylcholinesterase assays

Acetylcholinesterase activity of homogenate was spectrophotometrically measured by the method of Ellman [30] and Ikezawa and Taguchi [31] using the substrate of acetylthiocholine iodide. Each aliquot of whole body homogenate of adult (100 μL) was mixed with 800 μL of sodium phosphate buffer (pH 7.5), 50 μL of 10 mM DTNB reagent and 50 μL of 12.5 mM acetylthiocholine iodide. After the incubation period for 5 min at room temperature, the optical density of the sample was read at 400 nm using spectrophotometer (Lark LI-UV-2500) against suitable reagent blank.

### Carboxylesterase assays

The activity of α and β carboxylesterase activity of the whole body homogenate of adult was detected by the method of Asperen [32] and Koodalingam et al. [33] with slight modification. The 100 μL of homogenate was incubated with 900 μL of sodium phosphate buffer (20 mM pH 7.0) containing 250 μM of α- or β- naphthyl acetate for 30 min at room temperature. After incubation, 400 μL of 0.3% fast blue B in 3.3% SDS were added to stop the enzymatic reaction and incubated further for 15 min at room temperature. The optical density of the sample was read at 588 nm in spectrophotometer against the respective blank reagent.

### Acid and alkaline phosphatases assay

Acid and alkaline phosphatase levels of whole body homogenate of adult were measured by the method of Asakura [34] with suitable modification. The acid phosphatase activity was measured by adding of 50 μL of homogenate with 450 μL of 50 mM Sodium acetate buffer (pH 4.0) and 500 μL of 15 mM *p*-nitrophenyl phosphate. It was incubated, in water bath at 37°C for 15 min.

The alkaline phosphatase level in the homogenate was measured by adding 50 μL of homogenate with 450 μl of 50 mM Tris-HCl buffer (pH 8.0) and 500 μl of 15 mM *p*-nitrophenyl phosphate and incubated in water bath for 15 min at 37°C. Both the acid and alkaline phosphatase reactions were stopped by adding of 100 μL of 0.5 N NaOH solution and centrifuged (Refrigerated Centrifuge—SIGMA 1-15K, Germany) at 3000 rpm for 5 min and optical density of the sample was measured at 440 nm against suitable blank.

### Glutathione-S-Transferase activity

Glutathione-S-Transferase level of whole body homogenate of adult was measured by the method of Brogden and Barber [35]. 50 μL of homogenate was placed in microtiter plate. Then 200 μL of GSH/CDNB working solution was added. The plate was incubated for 10 min at room temperature and optical density of the sample was measured at 340 nm against suitable blank.

### Qualitative analysis of biochemical constituents

The profiles of protein, alpha and beta carboxylesterase of whole body homogenate of adult were analysed in non-denaturing condition by discontinuous polyacrylamide gel electrophoresis (PAGE). It was performed by using 4% stacking gel (pH 6.8) and 8% separating gel (pH 8.8) in Tris-glycine buffer (pH 8.3). Homogenate sample (50 μL) of treated and control adult was electrophoresed at 4 mA per sample and at 10°C. After electrophoresis, the gel was stained with suitable staining to detect the biochemical constituents. For protein profile, the gel was stained with Coomassie brilliant blue R-250 for the detection of bands.

#### Detection of α- and β- carboxylesterase activity

According to Kirkeby and Moe [36] and Argentine and James [37], the protein bands were detected from electrophoresed gel with alpha and beta carboxylesterase activity. For alpha/beta carboxylesterase, the gel was first incubated with phosphate buffer (20 mM, pH 7.0) for 15 min at room temperature. After decanting the buffer, the gel was re-incubated with freshly prepared α /β -naphthyl acetate + fast blue B solution for 30 min at room temperature. Then the gel was washed with double distilled water to remove excess stains from the gel and stored at 7% acetic acid.

#### Detection of acid or alkaline phosphatase activity

The gel was first incubated for 15 min at room temperature with 50 mM acetate buffer (pH 4.6) and 50 mM Tris-HCL buffer (pH 8.0) for acid and alkaline phosphatase activity respectively. After decanting the buffer, the gel was re-incubated with freshly prepared 50 mM acetate buffer (pH 4.6; contains α-naphthyl phosphate + fast blue B solution) and 50 mM Tris-HCL buffer (pH 8.0; contains α-naphthyl phosphate + fast blue B solution) for acid and alkaline phosphatase activity respectively for 2 hrs at 37°C on water bath [38]. Then the gels were washed with double distilled water to remove excess stains and stored at 7% acetic acid.

### Statistical analysis

Statistical analyses were done using the original data. The probit analysis was done for fumigation toxicity. The original data was subjected to homogeneity test (Levene Statistic) and normality test (Shapiro–Wilk test); based on the results, parametric test (Tukeys test), and non-parametric test (Kruskal Wallis test) were used. Significant difference between the treatment groups was calculated by ANOVA. Parametric test (Tukey’s test) was used for number of eggs laid, ovicidal, egg hatchability and larval survival, Non-Parametric test (Kruskal wallis test) was used for fumigation toxicity, larvicidal activity, knockdown and adult emergence (F1 generation) and for repellency Chi-Square test was used by SPSS-20.

## Results

### Oil yield

Initially the oil was whitish in colour but later it turned into pale yellow. The yield of EO was 0.65% v/w.

### Chemical composition of EO

Chemical composition of *C*. *citrinus* EO was analysed by GC-MS and it identified 10 different compounds in varying quantities (ranging between 0.59 to 40.44%). The total composition of the compounds were 100%. Among the 10 compounds, eucalyptol represented the major constituent (40.44%), followed by linalool (27.35%), and α- Pinene (17.36%) (Table 1).

**Table 1:** Chemical Constituents and composition (%) of essential oil from *Callistemon citrinus*.

Peak No.	Chemical Constituents	Retention Time (min)	Area%
1	α.-Pinene	5.690	17.46
2	Camphene	5.955	0.59
3	β.-Pinene	6.412	5.83
4	β.-Myrcene	6.627	3.22
5	β.-Pinene	6.852	0.93
6	Eucalyptol	7.315	40.44
7	gamma.-Terpinene	7.738	2.32
8	.alpha.-Methyl-.alpha.-[4-methyl-3-pentenyl]oxiranemethanol	7.936	0.92
9	2-Carene	8.165	0.96
10	Linalool	8.419	27.34

### Fumigation toxicity study

At 160 μL/L EO showed 100% adult beetle mortality at 9 hrs of treatment. At the lowest concentration (40 μL/L), EO exhibited 50% mortality after 24 hrs of exposure, and there was a gradual increase in insect mortality while increasing concentration of EO (Table 2). Maximum larvicidal activity of 95.78% was observed at 320 μL/L concentration at 48 hrs after exposure period (Table 3). The lethal concentration (LC_50_) of EO against *T*. *castaneum* adults was 37.05 μL/L at 24 h of exposure and against larvae it was 144.31 μL/L at 48 h of exposure (Table 4). The fumigation toxicity of *C*. *citrinus* EO was concentration and time dependent against *T*. *castaneum* larvae, however, the larvae, appeared to be more tolerant to EO than adults.

**Table 2:** Fumigation toxicity (%) of essential oil from *C*. *citrinus* against *T*. *castaneum*-adult.

Concentrations (μL/L)	After Exposure (h)				
	3	6	9	12	24
40	0.0±0.0^a^	12.0±2.00^a^	30.0±3.16^a^	36.0±2.45^a^	50.0±1.75^a^
80	0.0±0.0^a^	16.0±2.45^a^	58.0±2.00^ab^	68.0±3.74^ab^	81.3±1.93^ab^
120	0.0±0.0^a^	30.0±3.16^ab^	72.0±3.74^ab^	82.0±2.00^ab^	91.6±2.12^ab^
160	8.36±3.74^b^	48.0±3.74^b^	100.0±0.00^b^	100.0±0.00^b^	100.0±0.00^b^
200	22.0 ±2.00^b^	64.0±2.45^b^	100.0±0.00^b^	100.0±0.00^b^	100.0±0.00^b^
ANOVA	**p≤0.04 from 40, 80 and 120 μL/L	**p≤0.05 from 40 & 80 μL/L * p≤0.05 from 40 μL/L	**p≤0.01 from 40 μL/L	**p≤0.01 from 40 μL/L	**p≤0.01 from 40 μL/L

Mean of five replication ± SE; within the column same alpha bête do not significant by Kruskal-Wallis Test

**Table 3:** Larvicidal activity (%) of essential oil from *C*. *citrinus* against *T*. *Castaneum*.

Concen- trations (μL/L)	After Exposure (hrs)						
	3	6	9	12	24	36	48
40	0.0±0.0	0.0±0.0^a^	2.0±2.00^a^	4.0±2.45^a^	6.0±2.45^a^	10.2±3.16^a^	16.9±2.38^a^
80	0.0±0.0	0.0±0.0^a^	4.0±2.45^a^	14.0±2.45^a^	18.2±3.62^a^	22.9±1.84^ab^	31.6±3.99^ab^
120	0.0±0.0	0.0±0.0^a^	6.0±2.45 ^abc^	18.0±3.74^bc^	22.4±1.93^bc^	26.9±3.69^b^	42.2±3.76^bc^
160	0.0±0.0	6.0±2.45^ab^	10.0±3.16^abcd^	22.0±2.00^bc^	26.2±3.77^bc^	31.1±2.87^bc^	50.9±3.07^c^
200	0.0±0.0	8.0±2.00^ab^	14.0±2.45^bcd^	26.0±2.45^bcd^	30.4±2.82^bce^	35.1±3.47^bcd^	55.3±3.85^c^
240	0.0±0.0	12.0±2.00^bc^	16.0±2.45^cde^	30.0±3.16^cde^	34.7±2.26^cd^	41.8±1.09^cd^	70.4±3.39^d^
280	0.0±0.0	14.0±2.45^bc^	20.0±3.16^de^	36.0±2.45^de^	40.7±2.66^de^	47.8±3.34^d^	80.9±2.06^d^
320	0.0±0.0	18.0±3.74^c^	26.0±2.45^e^	42.0±2.00^e^	49.1±2.51^e^	64.7±2.00^e^	95.8±2.59^e^
Anova	*Df = 39,	Df = 39, F-11.55, p≤0.00	Df = 39, F-10.23, p≤0.00,	Df = 39, F-21.39, p≤0.00	Df = 39, F-22.75, p≤0.00	Df = 39, F-34.24, p≤0.00	Df = 39, F-65.64, p≤0.00

*No homogeneity; Mean of five replication ± SE; within the column same alpha bête do not significant by Tukey Test Test (p ≤ 0.05).

**Table 4:** Lethal concentration of essential oil from *C*. *citrinus* against *T*. *castaneum* adult and larvae.

Stage of the insect	LC_50_	95%Feducial level		LC_90_ (μL/L)	95%Feducial level		Chi-square
		Lower	Upper		Lower	Upper	
	**(μL/L)24 hrs after treatment**						
**Adult**	37.05	14.09	51.02	102.82	89.70	123.41	3.74
	**48 hrs after treatment**						
**Larva**	144.31	123.32	162.85	321.62	289.44	369.08	11.01

### Repellency test

The maximum repellency (93.3%) was observed at 20 μL concentration after 24 hrs observation. Lowest concentration showed 30% repellent activity against *T*. *castaneum* larvae at 24 hrs (Table 3). The highest concentration exhibited time related repellent activity by Chi-Square test (χ^2^–72, df-36, p≤0.00). Other concentrations did not show significant activity.

A 100% adult repellent activity was observed at 20 μL concentration after 24 hrs through Y-arm olfactometer. The lower concentration of EO exhibited more than 31.1% repellent activity (Table 5). The results indicated that EO exhibited good repellent potential against both larvae and adults. There was no significant effect found between the treatments by Chi-Square test (χ^2^–36, df-27, p≤0.12).

**Table 5:** Repellent activity (%) of essential oil from *C*. *citrinus* against *T*. *Castaneum*.

Concentration (μl/L)	After Exposure (hrs)					Chi-Square test		
	2	4	6	12	24			
	Larvae					χ^2^	df	p value
5	24.3±2.9	24.3±2.9	25.3±3.6	26.3±3.9	30.5±3.1	23.29	28	0.72
10	40.9±2.1	41.4±1.7	41.4±1.7	42.4±1.6	45.6±3.0	15.67	16	0.48
15	61.4±1.3	61.8±1.1	63.2±2.0	63.2±2.0	64.5±1.9	18.46	20	0.56
20	70.2±1.3	74.4±1.6	81.7±1.7	82.1±1.5	93.3±3.1	72.00	36	0.00
Adult-repellent activity (%)	Concentration (μl/L)							
	**5**	**10**	**15**	**20**	**-**	χ^2^	**df**	**p value**
	31.1±1.95	64.8±1.6	89.1±1.4	100±0.0	-	36.00	27	0.12

Mean of five replication ± SE

### Fecundity and knock-down activity

The control beetle group laid 5.8 eggs per individual on an average. In EO treated group, concentrations of 20 and 30 μL/L showed 2.6 and 1.4 eggs. In terms of per cent reduction, 20 and 30 μL/L concentrations showed 55.17 and 75.86% reduction in fecundity, respectively. *C*. *citrinus* EO significantly reduced oviposition activity at 20 and 30 μL/L (Table 6) Knockdown effect was increased according to increasing concentration. Maximum knockdown activity of 35.5% was observed at 30 μL/L which was statistically significant from the control (Kruskal-Wallis).

**Table 6:** Oviposition deterrent (number of eggs/insect/day) activity of essential oil from *C*. *citrinus* on *T*. *Castaneum*.

Concentrations (μL/L)	#Number of Eggs laid	Knockdown (%)
Control	5.8±0.37^d^	0.0±0.0
5	4.6±0.51^d^	0.0±0.0
10	3.8±0.20^c^	5.5±0.49
20	2.6±0.24^b^	24.0±0.61
30	1.4±0.24^a^	35.5±0.93*
Anova	Df-24, F-26.14, p≤0.00	Kruskal-Wallis Test ** p≤0.00 (control & 5 μL/L) (5 μL/L)

Mean of five replication ± SE (n = 50); within the column same alpha bête do not significant by Kruskal-Wallis Test (p ≤ 0.05) (Knock down); Tukeys test (#)

### Growth effects

#### Ovicidal activity and egg hatchability

The ovicidal effect of *C*. *citrinus* EO was studied against *T*. *castaneum* at four different concentrations. Maximum ovicidal activity of 91.49% was observed at 30 μL/L concentration of EO. All the concentrations showed statistically (Df-24, F-16924.3, p≤0.00) different activity from the control (Table 7).

**Table 7:** Bioefficacy of essential oil from *C*. *citrinus* against different life stages of *T*. *Castaneum*.

Concentrations—(μL/L)	Ovicidal activity (%)	Egg hatchability (%)	Larval survival (%)	*Adult emergence of F1 generation (%)
Control	10.85±0.25^a^	89.15±0.25^e^	86.96±1.23^e^	80.58±1.15
5	43.45±0.23^b^	56.55±0.24^d^	70.67±0.57^d^	53.45±0.27
10	56.70±0.36^c^	43.29±0.36^c^	60.61±1.41^c^	36.14±0.90
20	78.69±0.15^d^	21.31±0.15^b^	51.04±1.22^b^	21.82±0.67
30	91.49±0.13^e^	8.51±0.13^a^	42.54±1.32^a^	5.15±2.10**^#^
ANOVA	Df-24, F-16924.3, p≤0.00	Df-24, F-16924.3, p≤0.00	Df-24, F-210.12, p≤0.00	Kruskal-Wallis Test ** p≤0.00 (control) #p≤0.013 (5 μL/L)

Mean of five replication ± SE; within the column same alpha bête do not significantly by Tukeys test (P ≤ 0.05)

The 5 μL/L concentration exhibited egg hatchability of 56.55% while the control exhibited 89.15% egg hatchability. The minimum egg hatchability of 8.51% was recorded at 30 μL/L concentration (Table 7). The treatment showed significant difference from the control by Tukeys test (p≤0.00).

#### Larval survival and adult emergence

Larval survival and adult emergence (F_1_ generation) were 86.96% and 80.58%, respectively in the control. In contrast, at 30 μL/L concentration EO exhibited larval survival of 42.54%, and it was notably lower than the control. Significant reduction of larval survival was recorded in all the treatments when compared to the control. The 30 μL/L treatment allowed 5.15% of adults to emerge when compared to the control. A significant reduction in adult emergence (F_1_) 5.15% was observed at 30 μL/L (Table 7). The 30 μL/L concentration was statistically significant from other treatments and the control.

### Quantitative analysis of biochemical constituents

Based on the obtained results, sub-lethal concentrations (5_,_ 10 and 20 μL/L) were used to study the impact of *C*. *citrinus* EO on various biochemical constituents in adult *T*. *castaneum* beetles. Results indicated that the biochemical constituents measured in *T*. *castaneum* adult beetles significantly varied after exposure to sub-lethal concentrations of EO for 24 hrs. The total protein content of *T*. *castaneum* adult was highly and significantly reduced 6.19 mg/mL, 5.72 mg/mL, 5.32 mg/mL in adults exposed to different concentrations of 5, 10 and 20 μL/L of EO, respectively relative to the control value of 7.43 mg/mL. The higher concentration was statistically different from other treatments and control. Acetylcholinesterase activity dramatically increased in the 10 and 20 μL/L_,_ treatments when compared to the control; while, at 20 μL/L concentration treatment, there was reduced Acetylcholinesterase activity. The highest concentration statistically and significantly influenced enzyme activity when compared to the control (Kruskal-Wallis Test p ≤ 0.028) (Table 8).

**Table 8:** Biochemical effect of essential oil of *C*. *citrinus* on *T*. *castaneum*.

BIOCHEMICAL STUDIES	Concentrations–(μL/L)				ANOVA
	Control	5	10	20	
Total protein (μg protein/μl of homogenate	7.43±0.07	6.19±0.03	5.72±0.15	5.32±0.21*	Kruskal-Wallis Test p≤0.022
Acetylcholinesterase (μM of AcT Hydrolysed/min/mg of protein)	3.30±0.04	3.68±0.05	3.74±0.04	2.93±0.06*	Kruskal-Wallis Test (p ≤ 0.039)
α-Carboxylesterase (μM of α-Naphthol released/min/mg of protein)	67.06±1.83	85.46±1.13	99.38±2.25	110.59±2.69*	Kruskal-Wallis Test (p ≤ 0.013)
β-carboxylesterase (μM of β-Naphthol released/min/mg of protein)	0.056±0.0002	0.137±0.0009	0.137±0.0007	0.133±0.0015	Kruskal-Wallis Test (p ≤ 0.055)
Acid-phosphatase (mM *p-*nitrophenol released/min/mg of protein)	0.86±0.02	0.84±0.04	0.73±0.10	0.60±0.11	Tukeys Test (p ≤ 0.137)
Alkaline-phosphatase (mM *p-*nitrophenol released/min/mg of protein)	0.42±0.02	0.30±0.03	0.71±0.06	0.14±0.04*	Kruskal-Wallis Test (p ≤ 0.013)
**Glutathione-S-Transferase (μM of GS-CDNB conjugated/min/mg of protein)**	0.219±0.002	0.365±0.003	0.333±0.001	0.376±0.009	**Kruskal-Wallis Test (p ≤ 0.027)**

* Significant from control

The level of α-Carboxylesterase activity was significantly increased at three of the selected sub-lethal concentrations of EO, compared to the control, but statistically significant activity was found at 20 μL/L. β-carboxylesterase activity level was also drastically elevated in the 5, 10 and 20 μL/L treatments compared to the control; however, no significant difference was observed between the different sub-lethal treatments (Table 8).

Exposure of *T*. *castaneum* adults to *C*. *citrinus* EO resulted in decreased level of acid phosphatase activity at selected concentrations when compared to the control group. Alkaline phosphatase activity was significantly reduced in 5 μL/L treatment and drastically elevated in the 10 μL/L treatment. Significantly lower activity was recorded in the 20 μL/L treatment when compared to the control. Glutathione-S-Transferase levels gradually increased in the 5, 10 and 20 μL/L treatments, relative to the control (Table 8).

### Qualitative analysis of biochemical constituents

**Fig 1:**
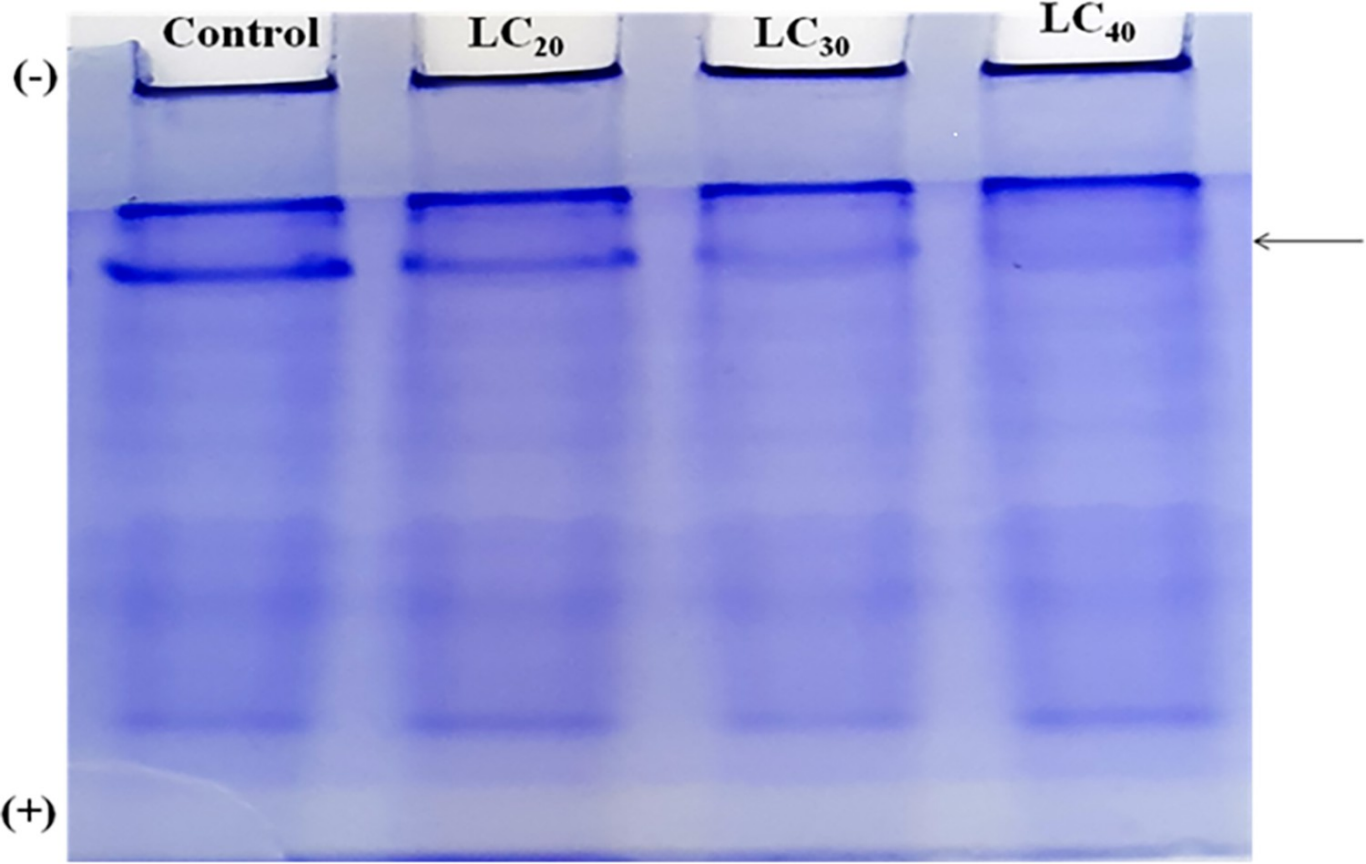
Qualitative analysis of total protein in native PAGE, of *T*. *castaneum* adult after treatment with essential oil of *C*. *Citrinus*.

**Fig 2:**
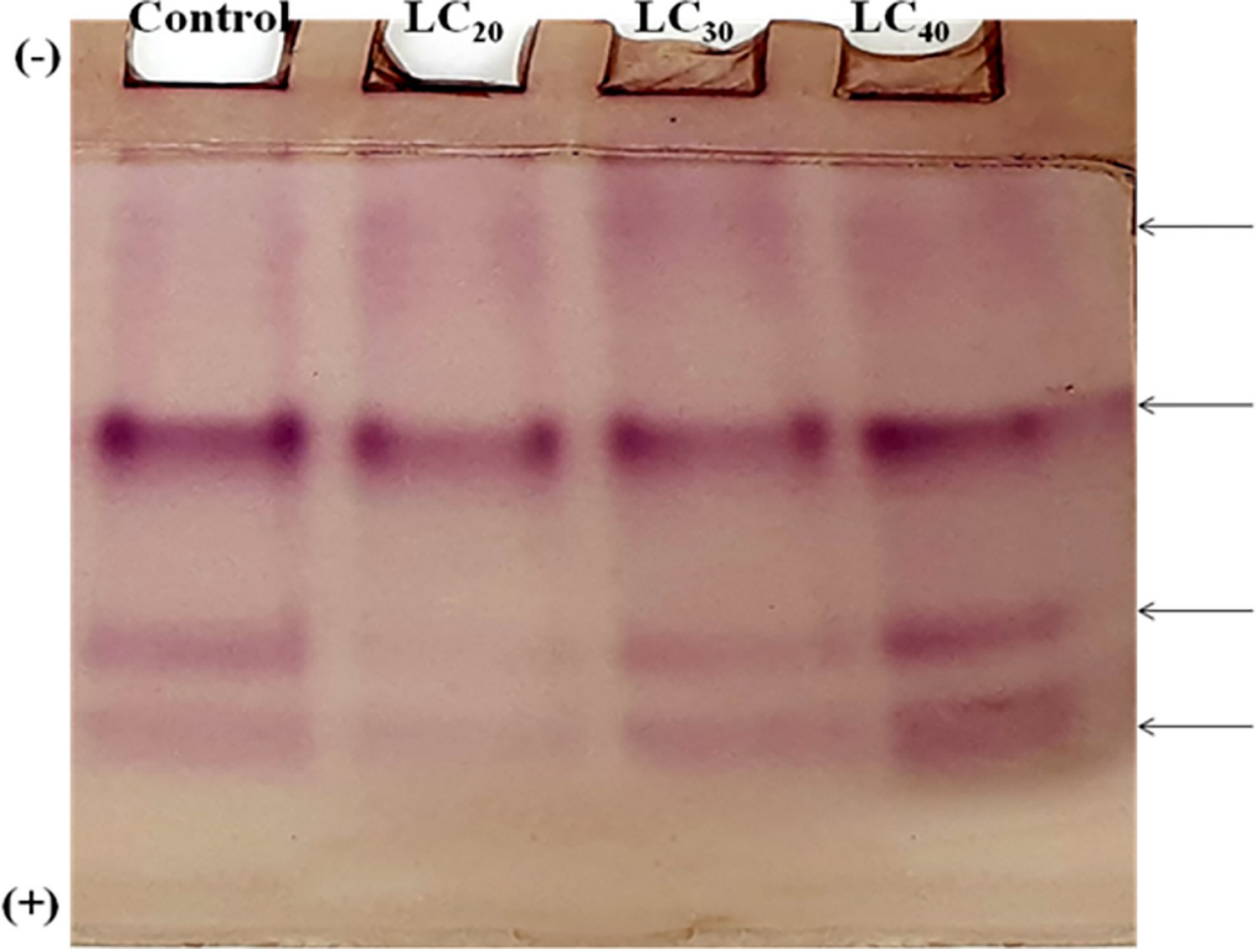
Qualitative analysis of isoenzyme of β-Carboxylesterases of *T*. *castaneum* adult after treatment with essential oil of *C*. *Citrinus*.

**Fig 3:**
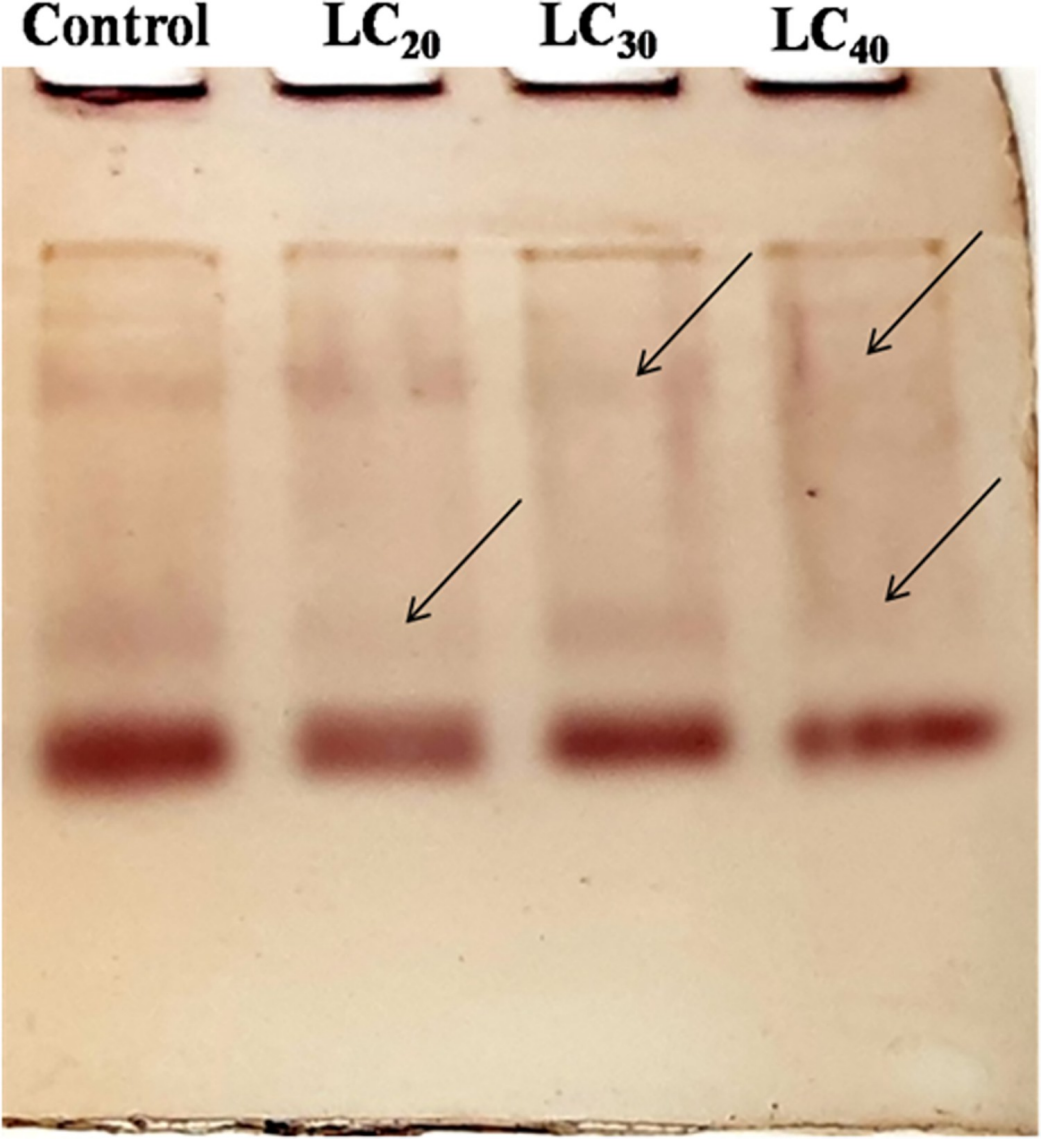
Qualitative analysis of acid phosphatises of *T*. *castaneum* adult after treatment with essential oil of *C*. *Citrons*.

**Fig 4:**
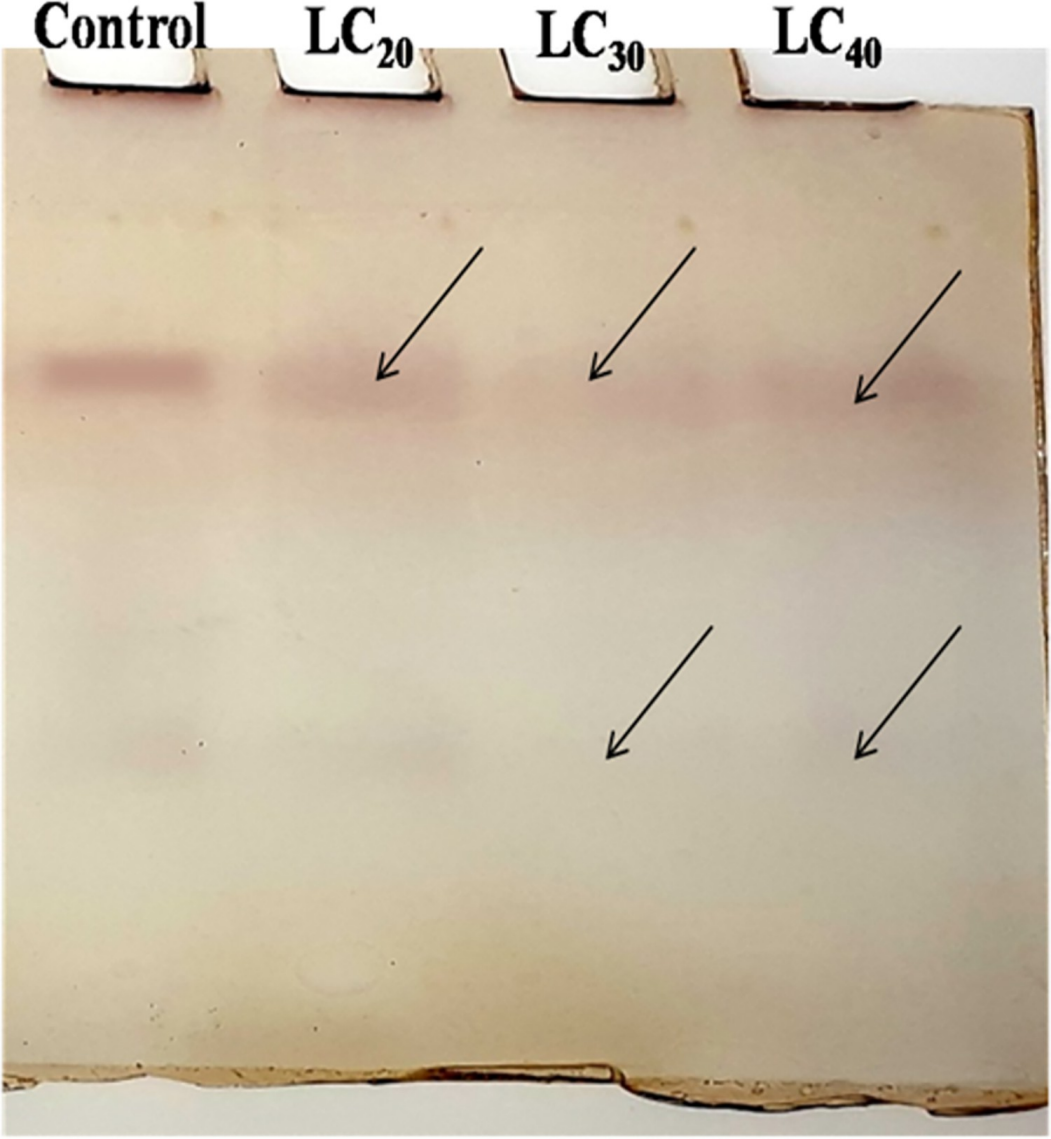
Qualitative analysis of alkaline phosphatases *T*. *castaneum* adult after treatment with essential oil of *C*. *Citrinus*.

A qualitative analysis of total proteins was done using the native PAGE method. Protein extracted from *T*. *castaneum* adults treated with sub-lethal concentrations of *C*. *citrinus* EO showed a reduction in the number protein bands, compared to the control (Fig 1). The intensity of the esterase band of β-Carboxylesterase isoenzyme was modulated by the concentration of EO. The intensity of the band was lowered in the treatment but increased gradually as the concentration of EO increased. The two lower isoenzyme bands decreased in their intensity, relative to the control, at the lower concentrations of EO and gradually increased when beetles were exposed to higher concentration of EO (Fig 2). Electrophoretic analysis of acid and alkaline phosphatase enzyme activity in adult beetles was slightly affected by exposure to the range of sub-lethal concentrations of EO used in the experiment (Figs 3 and 4).

## Discussion

Botanical pesticides are generally biodegradable, and have the potential to eradicate pests without causing harm to the environment and non-target organisms. Essential oils are volatile in nature; they are easy to use and kill the pests of stored grains. Hence, in the present study, EO extracted from *C*. *citrinus* leaves was tested for its potential against the larvae and adult beetles of *T*. *castaneum*.

GC-MS analysis of *C*. *citrinus* revealed ten different compounds, in which eucalyptol (40.44%) was the major constituent, followed by linalool, and α- Pinene. However, the same plant from Ethiopia contained 76.9% of eucalyptol and from Western Himalayas it contained only 9.8% eucalyptol [39, 40]. These data clearly indicate that the level and type of constituents in EO extracted from this plant varies depending on where the plant was collected, and most likely the climate and ecology of that region playing a role. These results are in accordance with Misharina [41], Souza and Vendramim [42] and Isikber et al. [43] who indicated that the variations in extracted compounds, could be associated with geographical location, collection time, amount of sunlight, length of storage, temperature, and extraction methods.

Fumigation is one of the most effective, practically feasible, and rapid methods that can be used to protect feedstuffs, stored food grains and other agricultural products from pest infestation [44, 45]. In the present study, fumigation activity of EO extracted from leaves of *C*. *citrinus* showed 100% mortality in adult beetles of *T*. *castaneum* at 9 hrs after treatment at a concentration of 160 μL/L. Similar results were obtained from *Coriandrum sativum* seed oil, where mortality in *C*. *maculatus* and *T*. *confusum* increased with increasing concentrations of EO from 43 to 357 *μ*L/L air [46]. The EO used in our study exhibited different levels of toxicity to larvae vs. adult beetles. In general, fumigation toxicity was lower against larvae than adult beetles. Earlier, Huang et al. [47] and Isikber [43] have reported similar results.

In the present study, a significant reduction in oviposition was recorded when adult beetles were exposed to different concentrations of EO. Similarly, Moura et al. [20] also reported that EO derived from *Vanillosn opsis arborea* reduced the level of oviposition when compared to the control. A previous report indicated decreased oviposition (28%) at 5.2 mg/cm^2^ concentration when the adult beetles were exposed to EO derived from leaves of *C*. *loga*. The reduction in oviposition was probably due to physically weakened insects as well as lesser surviving insects [48].

Egg hatchability was drastically reduced because the vapour of EO diffused through the permeable membranes of insect eggs into the chorion and vitelline membrane [49, 50]. The diffusion of EO vapours into the eggs results in a disruption in normal physiological and biochemical processes [51]. The ovicidal activity observed in the present study confirmed the above statements, as the tested EO resulted in 91.49% ovicidal activity in *T*. *castaneum*. The reduction of the F1 generation could be due to the toxicity of EO to all life stages of the insect, from eggs to adults, *via*., both fumigant and possibly by stomach action [52]. In the present study, a drastic reduction (94.85%) was observed in adult emergence when the eggs were exposed to EO.

Proteins are the most abundant organic compounds in the insect body as they provide structure, energy and catalyse chemical reactions in the form of enzymes [53, 54]. Decreasing protein content commonly occurs when the insects are treated with lethal compounds [55]. Insects degrade the protein content into amino acid and release energy to compensate the lowering energy level during stress condition [56]. Reductions in protein levels were observed in the present study and has been previously reported that protein content was reduced due to the toxicity of plant products [27, 57–59].

In the present study, AChE activity was inhibited at the higher concentrations of *C*. *citrinus* oil. Saponins were able to inhibit AChE and the inhibition increased with increasing concentration [60]. Inhibition of AChE results in the accumulation of acetylcholine at cholinergic synapses and causes hyper excitation of cholinergic pathways [61].

Carboxylesterase activity can be altered by plant secondary metabolites. Phenolic glycoside significantly increased the level of Carboxylesterase in *Lymantria dispar* [62]. A higher level of CarE activity was recorded in *Sitobion avenae* fed on diet with high indole alkaloid content [63]. In the present study, both α- CarE and β- CarE levels increased in adult beetles exposed to *C*. *citrinus* EO.

Hydrolytic cleavage of phosphoric acid esters is catalysed by Phosphatase enzymes that are classified into "acid" or "alkaline" phosphatases based on their pH [64]. Acid phosphatases are a lysosomal marker enzyme whose active site is in the gut of insects [65–68]. Alkaline phosphatases are a brush border membrane marker [69]. Exposure to plant compounds reduced the acid and alkaline phosphatises content in *Cnaphalocrocis medinalis* larvae [70, 71]. Similarly, *C*. *citrinus* EO also reduced both acid and alkaline phosphatase activity in a concentration dependent manner.

Glutathione S-transferases are the enzymes that catalyse the detoxification of insecticides typically after the phase-I metabolic process [72]. In the present study, elevated GST levels were observed in adult beetles exposed to the higher concentrations of *C*. *citrinus* EO. Shojaei et al. [73] reported that adults of *T*. *castaneum* exposed to *Artemisia dracunculus* EO enhanced the level of Glutathione-S-Transferase in a concentration dependent manner.

The present study clearly indicated that the EO possessed wide range of biological activities which included fumigant, repellent, oviposition and growth inhibitory activities, and acted up on all insect development stages. Hence, this EO should be prepared as a formulation and further regulatory studies (ecotoxicological effects, genetic toxicological effects and acute toxicity studies along with stability studies) should be conducted for safety assessment. Then it could be considered for used in stored product pest management.

## Supporting information

S1 File Y-Tube Suplimentary file. (PPTX)

S2 File Supporting data. (PPTX)

S1 Data (PDF)
