# Supplementary material for: Essential oil composition of Callistemon citrinus (Curtis) and its protective efficacy against Tribolium castaneum (Herbst) (Coleoptera: Tenebrionidae)
Source: PLoS One. 2022 Aug 19;17(8):e0270084. doi: 10.1371/journal.pone.0270084 (PMC9390898; doi:10.1371/journal.pone.0270084)
Supplement: S1 File — (PPTX) [file pone.0270084.s001.pptx]

## Slide 1
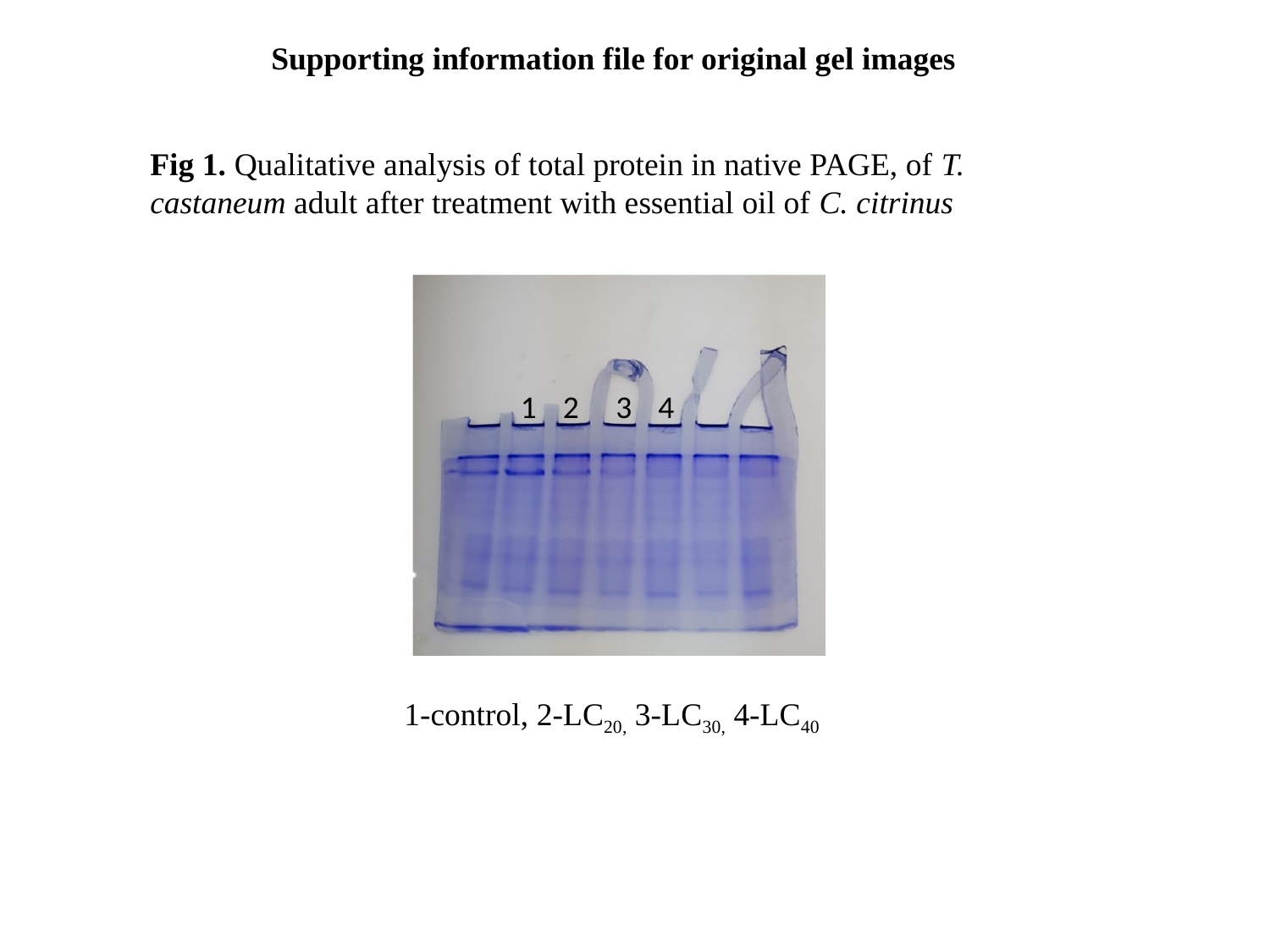

Supporting information file for original gel images
Fig 1. Qualitative analysis of total protein in native PAGE, of T. castaneum adult after treatment with essential oil of C. citrinus
1
2
3
4
1-control, 2-LC20, 3-LC30, 4-LC40

## Slide 2
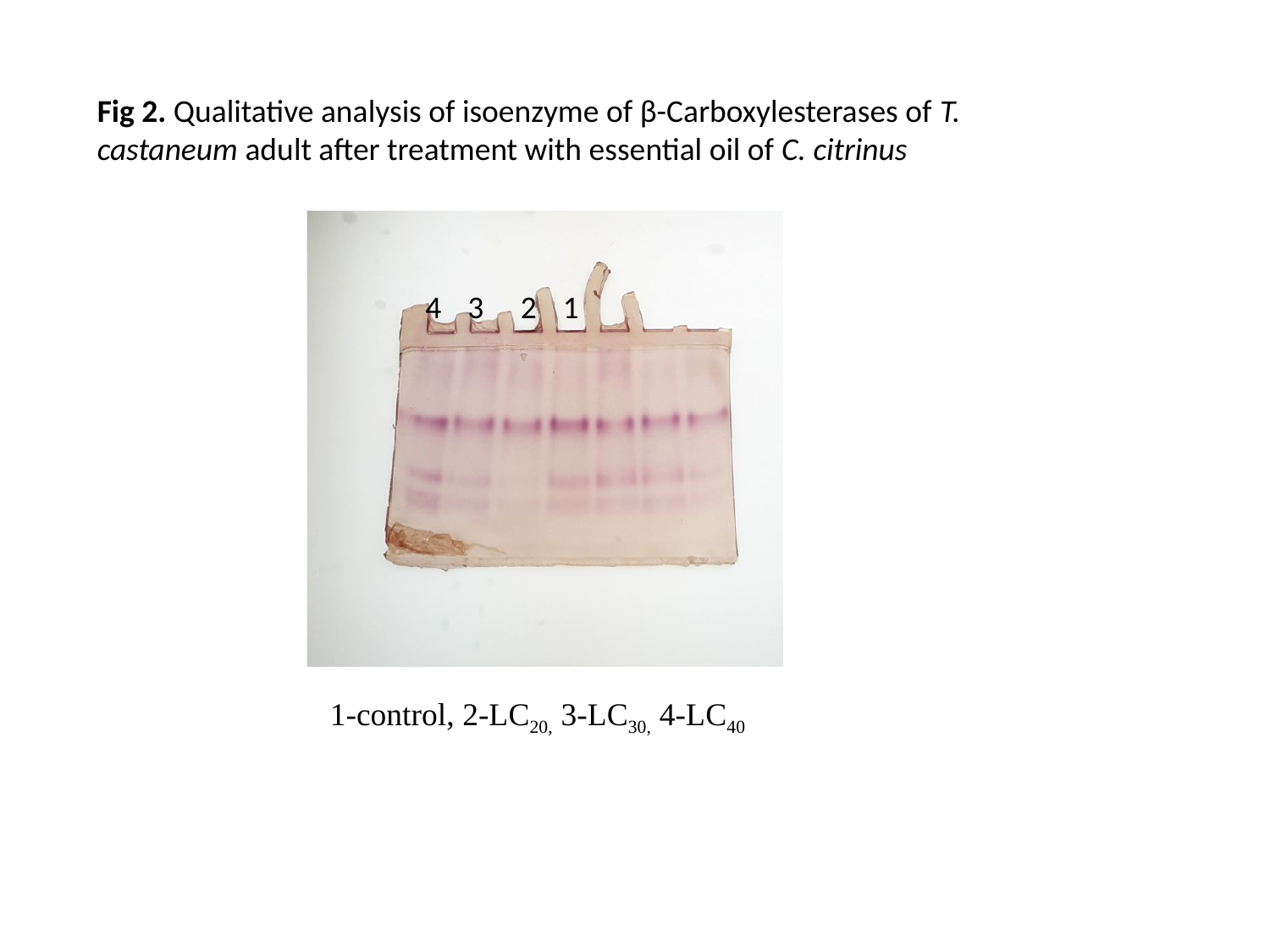

Fig 2. Qualitative analysis of isoenzyme of β-Carboxylesterases of T. castaneum adult after treatment with essential oil of C. citrinus
4
3
2
1
1-control, 2-LC20, 3-LC30, 4-LC40

## Slide 3
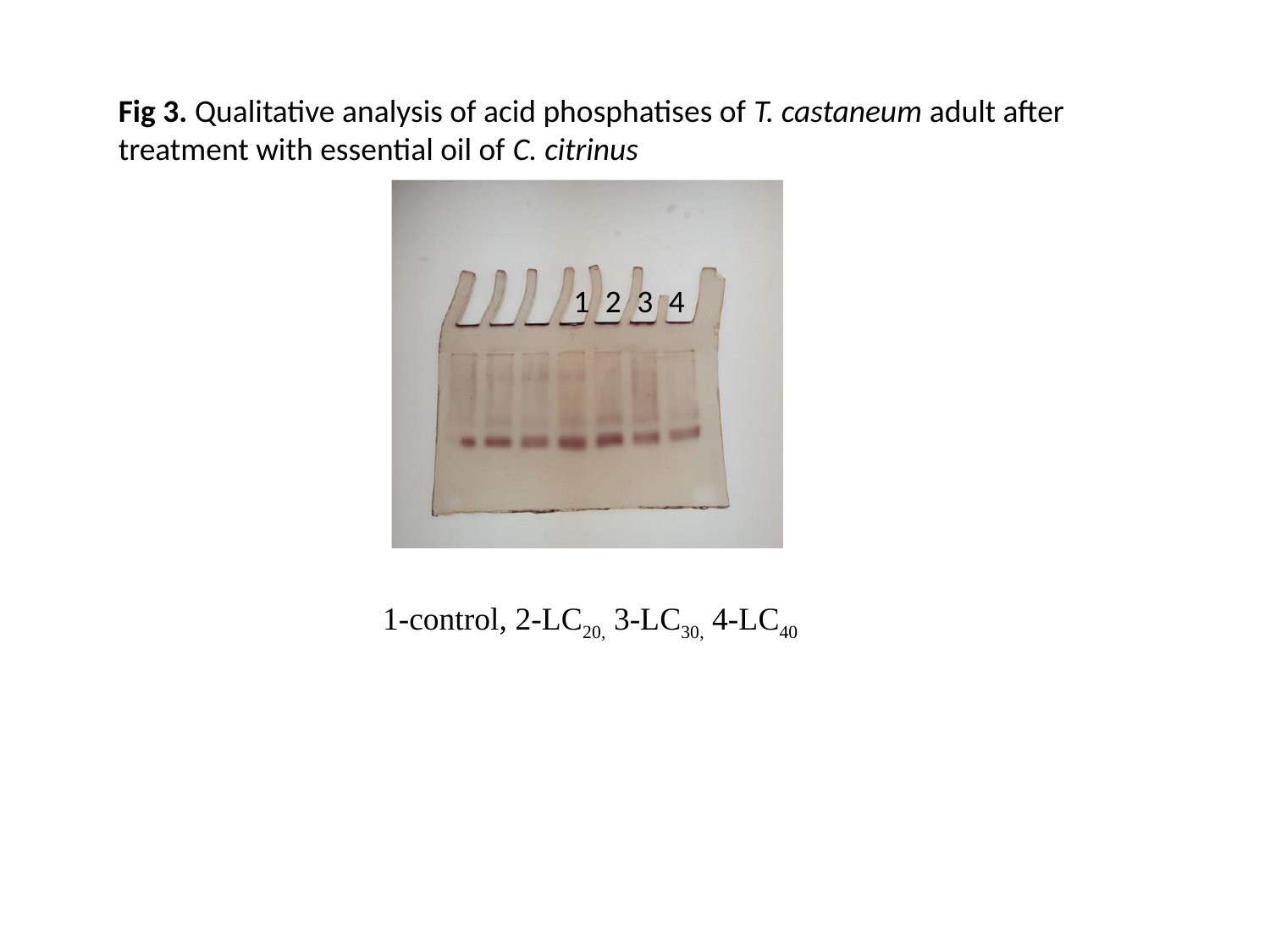

Fig 3. Qualitative analysis of acid phosphatises of T. castaneum adult after treatment with essential oil of C. citrinus
1
2
3
4
1-control, 2-LC20, 3-LC30, 4-LC40

## Slide 4
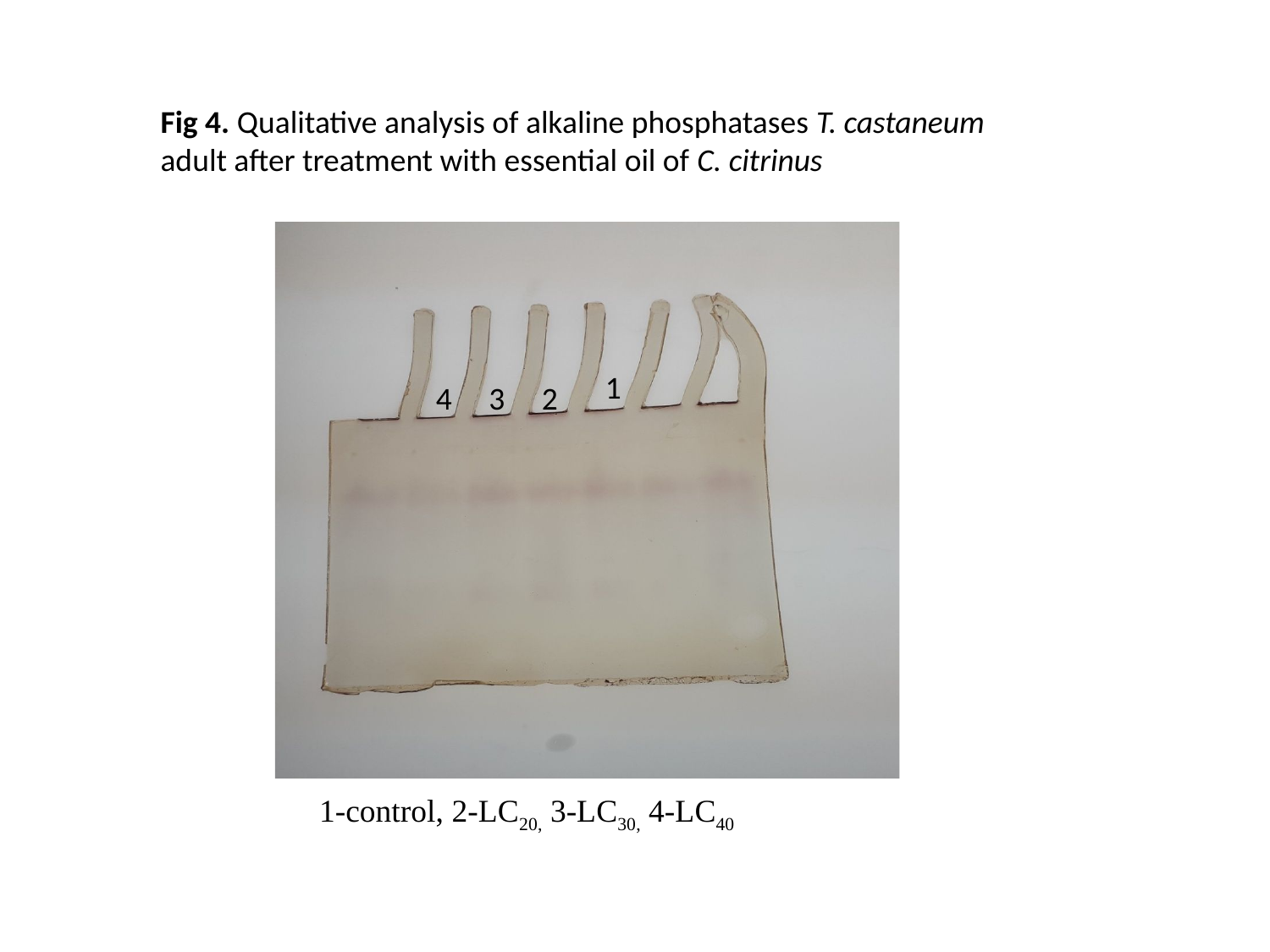

Fig 4. Qualitative analysis of alkaline phosphatases T. castaneum adult after treatment with essential oil of C. citrinus
1
4
3
2
1-control, 2-LC20, 3-LC30, 4-LC40
