# Supplementary figures and images for: Essential oil composition of Callistemon citrinus (Curtis) and its protective efficacy against Tribolium castaneum (Herbst) (Coleoptera: Tenebrionidae)
Source: PLoS One. 2022 Aug 19;17(8):e0270084. doi: 10.1371/journal.pone.0270084 (PMC9390898; doi:10.1371/journal.pone.0270084)

## Slide 1
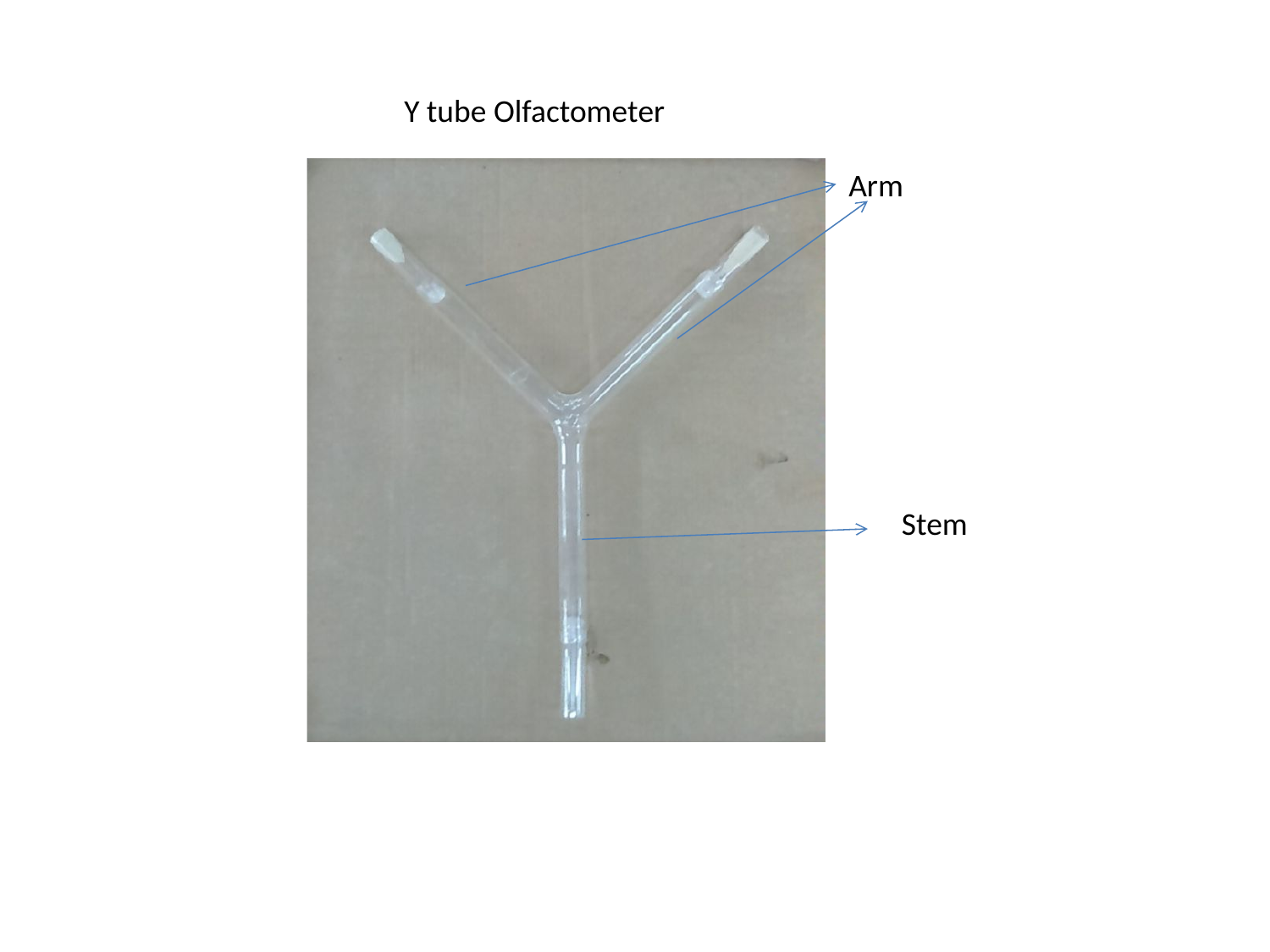

Y tube Olfactometer
Arm
Stem

Supplement: S2 File — (PPTX) [file pone.0270084.s002.pptx]
