# Supplementary material for: Essential oil composition of Callistemon citrinus (Curtis) and its protective efficacy against Tribolium castaneum (Herbst) (Coleoptera: Tenebrionidae)
Source: PLoS One. 2022 Aug 19;17(8):e0270084. doi: 10.1371/journal.pone.0270084 (PMC9390898; doi:10.1371/journal.pone.0270084)
Supplement: S1 Data — (PDF) [file pone.0270084.s003.pdf]

6/7/2018

Bioassay : Fumigation Toxicity

Target pest : Tribolium castaneum adult

Treatment source : Callistemon citrinus essential oil

Concentrations : 40µl/L, 80µl/L, 120µl/L, 160µl/L, 200µl/L

Study period : 3h, 6h, 9h, 12h, 24h

Replication : 5

No. of Insect/Replicate : 10.

Concentrations & Replicates		Insect Mortality – Exposure Time				
		3h	6h	9h	12h	24h
40µl/L	R_1	0	1	2	4	5
	R_2	0	2	4	4	5
	R_3	0	1	3	3	5
	R_4	0	1	3	3	5
	R_5	0	1	3	4	6

Done BY
M. RAMACHANDRAN
M. R[illegible] (signature)

Confirmed......
[signature] 8/7/2018.

		3h	6h	9h	12h	24h
80µl/L	R1	0	1	5	8	9
	R2	0	2	6	6	8
	R3	0	2	6	7	8
	R4	0	1	6	6	8
	R5	0	2	6	7	8
120µl/L	R1	0	3	8	8	9
	R2	0	4	7	8	9
	R3	0	2	8	8	9
	R4	0	3	6	9	10
	R5	0	3	7	8	9

Done By
M. RAMACHANDRAN
M.R. [illegible]

Continued ......
[illegible]
8/7/2018.

		3h	6h	9h	12h	24h
160µl/L	R1	2	5	10	10	10
	R2	1	4	10	10	10
	R3	1	6	10	10	10
	R4	0	4	10	10	10
	R5	0	5	10	10	10
200µl/L	R1	2	7	10	10	10
	R2	3	7	10	10	10
	R3	2	6	10	10	10
	R4	2	6	10	10	10
	R5	2	6	10	10	10
Control	R1	0	0	0	0	1
	R2	0	0	0	0	0
	R3	0	0	0	0	0
	R4	0	0	0	0	0
	R5	0	0	0	0	1

Done BY
M. RAMACHANDRAN
M.R[illegible]

[illegible] 8/7/2018

Page 1-3: Minimal data for adult fumigation toxicity - for Manuscript Table 2

18/7/18

Bioassay : Fumigation Toxicity

Target Pest : Tribolium castaneum Larvae

Treatment source : Callistemon citrinus essential oil.

concentrations : 40µl/L, 80µl/L, 120µl/L, 160µl/L, 200µl/L, 240µl/L, 280µl/L, 320µl/L.

Study period : 3h, 6h, 9h, 12h, 24h, 36h, 48h

Replication : 5

No. of Insect/Replicate : 10.

Concentration & Replication		Larval mortality						
		Exposure Time						
		3h	6h	9h	12h	24h	36h	48h
40µl/L	R_1	0	0	0	0	1	2	2
	R_2	0	0	1	1	1	1	3
	R_3	0	0	0	1	1	1	2
	R_4	0	0	0	0	0	1	2
	R_5	0	0	0	0	1	2	2

Done BY
M.RAMACHANDRAN
M.R[illegible]

Continued......

[illegible] 2/7/2018

Concentration	Replicate	3h	6h	9h	12h	24h	36h	48h
80µl/L	R1	0	0	0	1	1	2	4
	R2	0	0	1	2	2	3	3
	R3	0	0	1	1	2	2	4
	R4	0	0	0	1	2	3	4
	R5	0	0	0	2	3	3	3
120µl/L	R1	0	0	0	3	3	4	5
	R2	0	0	1	2	3	3	4
	R3	0	0	1	2	2	2	5
	R4	0	0	1	1	2	3	5
	R5	0	0	0	1	2	3	4
160µl/L	R1	0	1	2	2	3	4	5
	R2	0	0	1	2	2	3	5
	R3	0	1	1	2	3	3	6
	R4	0	1	1	2	3	3	5
	R5	0	0	0	3	3	4	6
200µl/L	R1	0	1	2	2	4	4	6
	R2	0	1	1	2	3	3	5
	R3	0	1	1	3	3	4	5
	R4	0	1	1	3	3	4	6
	R5	0	1	2	3	3	4	7

Done BY
M. RAMACHANDRAN
M. [illegible]

Continued.... [illegible] 2/7/2018

		3h	6h	9h	12h	24h	36h	48 h
240µl/L	R1	0	2	2	4	4	4	8
	R2	0	1	1	3	4	5	7
	R3	0	1	2	3	3	4	6
	R4	0	1	1	2	4	4	7
	R5	0	1	2	3	3	5	8
280µl/L	R1	0	2	2	4	4	5	9
	R2	0	1	1	3	4	5	8
	R3	0	2	2	4	4	4	8
	R4	0	1	2	4	5	6	8
	R5	0	1	3	3	4	5	8
320µl/L	R1	0	2	3	4	5	7	10
	R2	0	1	2	4	6	7	9
	R3	0	3	3	5	5	6	10
	R4	0	1	3	4	4	6	9
	R5	0	2	2	4	5	7	10
Control	R1	0	0	0	0	0	0	1
	R2	0	0	0	0	1	1	1
	R3	0	0	0	0	0	0	0
	R4	0	0	0	0	0	0	0
	R5	0	0	0	0	0	1	1

Done BY
M. RAMACHANDRAN
M.R[illegible]

[illegible signature]
21/7/2018.

Page 4-6: Minimal data for Larval fumigation toxicity - for Manuscript Table 3

24/7/18.

Bioassay : Repellent activity (Y-tube) ✓

Target Pest : Tribolium castaneum adult ✓

Treatment source : Callistemon citrinus essential oil

Concentrations : 5µl, 10µl, 15µl, 20µl ✓

Study Period : 24h.

Replication : 3

No. of insect /Replicate : 50

Exposure Time & Concentrations		Adult Repellency					
		Replication					
		R_1		R_2		R_3	
		T	C	T	C	T	C
24h	5µl	16	33	17	32	18	32
	10µl	9	38	8	40	8	39
	15µl	3	45	2	47	3	47
	20µl	0	45	0	47	0	49

Done BY
M. RAMACHANDRAN
M.R.M.

[signature] 20/08/2018

Page 7: Minimal data for adult repellent activity - for Manuscript Table 5

21/8/18

Bioassay : Repellent activity (Choice-based method)

Target pest : Tribolium castaneum Larvae

Treatment source : Callistemon Citrinus essential oil

Concentrations : 5µl, 10µl, 15µl, 20µl

Study period : 2h, 4h, 6h, 12h, 24h.

Replication : 5

No.of insect/Replicate : 25.

Concentration & Replicate		Larval Repellency Exposure Time									
		2h		4h		6h		12h		24h	
		T	C	T	C	T	C	T	C	T	C
5µl	R1	9	16	9	16	9	16	9	16	8	16
	R2	9	16	9	16	9	16	8	16	7	16
	R3	8	14	8	14	7	14	7	14	7	14
	R4	9	15	9	15	9	15	9	15	9	15
	R5	10	13	10	13	11	14	11	14	9	14

Done BY
M. RAMACHANDRAN
M.R. [signature]

Continued - - -
[signature] 22/08/2018

		2h		4h		6h		12h		24h	
		T	C	T	C	T	C	T	C	T	C
10d	R1	7	18	7	18	7	18	7	18	6	19
	R2	7	18	7	18	7	18	7	18	6	19
	R3	7	18	7	18	7	18	7	18	7	18
	R4	7	16	7	16	7	16	7	18	7	18
	R5	8	16	8	17	8	17	8	17	8	17
15d	R1	5	20	5	20	5	20	4	21	4	21
	R2	5	20	5	20	5	20	4	21	4	21
	R3	5	19	5	20	5	20	5	20	5	20
	R4	4	19	4	19	4	21	5	20	4	20
	R5	4	18	4	18	4	21	5	20	5	20
20d	R1	3	18	3	22	2	22	2	23	0	23
	R2	3	18	3	22	2	22	2	22	2	22
	R3	4	19	4	21	3	21	3	22	0	22
	R4	3	18	3	22	2	22	2	23	1	23
	R5	3	18	3	22	2	22	2	22	1	22

Done BY
M. RAMACHANDRAN
M.R.M.

[illegible] 22/08/2018

Page 8-9: Minimal data for larval repellent activity - for Manuscript Table 5
